# Evidence for Extensive Duplication and Subfunctionalization of *FCRL6* in Armadillo (*Dasypus novemcinctus*)

**DOI:** 10.3390/ijms24054531

**Published:** 2023-02-25

**Authors:** Maria Carolina Matos, Ana Pinheiro, Randall S. Davis, Pedro J. Esteves

**Affiliations:** 1 CIBIO-UP, Centro de Investigação em Biodiversidade e Recursos Genéticos, InBIO Laboratório Associado, Campus Agrário de Vairão, Universidade do Porto, 4485-661 Vairão, Portugal; 2 BIOPOLIS Program in Genomics, Biodiversity and Land Planning, CIBIO, 4485-661 Vairão, Portugal; 3 Departamento de Biologia, Faculdade de Ciências, Universidade do Porto, 4169-007 Porto, Portugal; 4 Departments of Medicine, Microbiology, and Biochemistry & Molecular Genetics, Comprehensive Cancer Center, University of Alabama at Birmingham, Birmingham, AL 35294, USA; 5 CITS-Centro de Investigação em Tecnologias de Saúde, Cooperativa de Ensino Superior Politécnico e Universitário, CRL (CESPU), 4585-116 Gandra, Portugal

**Keywords:** *FCRL*, *FCRL6*, armadillo, gene duplication, subfunctionalization, leprosy

## Abstract

The control of infections by the vertebrate adaptive immune system requires careful modulation to optimize defense and minimize harm to the host. The Fc receptor-like (*FCRL*) genes encode immunoregulatory molecules homologous to the receptors for the Fc portion of immunoglobulin (FCR). To date, nine different genes (*FCRL1–6*, *FCRLA*, *FCRLB* and *FCRLS*) have been identified in mammalian organisms. *FCRL6* is located at a separate chromosomal position from the *FCRL1-5* locus, has conserved synteny in mammals and is situated between the *SLAMF8* and *DUSP23* genes. Here, we show that this three gene block underwent repeated duplication in *Dasypus novemcinctus* (nine-banded armadillo) resulting in six *FCRL6* copies, of which five appear functional. Among 21 mammalian genomes analyzed, this expansion was unique to *D. novemcinctus*. Ig-like domains that derive from the five clustered *FCRL6* functional gene copies show high structural conservation and sequence identity. However, the presence of multiple non-synonymous amino acid changes that would diversify individual receptor function has led to the hypothesis that *FCRL6* endured subfunctionalization during evolution in *D. novemcinctus*. Interestingly, *D. novemcinctus* is noteworthy for its natural resistance to the *Mycobacterium leprae* pathogen that causes leprosy. Because *FCRL6* is chiefly expressed by cytotoxic T and NK cells, which are important in cellular defense responses against *M. leprae*, we speculate that *FCRL6* subfunctionalization could be relevant for the adaptation of *D. novemcinctus* to leprosy. These findings highlight the species-specific diversification of *FCRL* family members and the genetic complexity underlying evolving multigene families critical for modulating adaptive immune protection.

## 1. Introduction

Adaptive immunity provides the core defense mechanism for controlling infections in vertebrates. However, antigen-specific responses require careful regulation to ensure that pathogens are neutralized without inadvertently harming the host [1]. The buffering and fine-tuning of adaptive responses is largely mediated by immunoregulatory molecules encoded by receptor-gene families that can promote and/or suppress reactivity. One example is the Fc receptor-like (*FCRL)* genes, which are linked by common ancestry to the IgG and IgE-binding classical Fc receptors (*FCR*) [1,2]. The *FCR* and the *FCRL* families not only share a similar structure and genetic organization, but also related extracellular Ig-like domains and an amino-terminal split signal peptide that consists of a 21 bp second exon that encodes the second half of the leader sequence [1,2,3,4]. Nine *FCRL* genes have been identified in mammalian genomes (*FCRL1–6*, *FCRLA*, *FCRLB* and *FCRLS*); however, not all are simultaneously present in all species. Based on human and mouse studies, most encode type I transmembrane glycoproteins that are restricted to B cells, with the exception of *FCRL6* and *FCRLS*, which are expressed among different lymphocyte and myeloid lineages [5,6]. Despite their ectodomain similarity with the classical FCRs, the ligand binding properties of human *FCRLs* differ [1,3]. For example, secretory IgA is a ligand for FCRL3 [7], systemic IgA for FCRL4 [8,9], IgG for FCRL5 [9,10] and HLA/MHC class II molecules for FCRL6 [11]. The complexity of the FCRL family is underscored by genetic polymorphisms, multiple splice isoforms and tyrosine-based signaling features. While some family members have cytoplasmic sequences resembling immunoreceptor tyrosine-based activation motifs (ITAM), based on the high prevalence of immunoreceptor tyrosine-based inhibitory motifs (ITIM), FCRL1–6 appear to preferentially dampen immune responses [1,5]. Given their regulatory properties and lymphocyte-restricted distribution, there has been increasing interest in the associations of *FCRL*s with lymphoproliferative disorders, autoimmune diseases and immunodeficiencies [1,3,12,13,14,15,16,17]. These growing relationships collectively imply potential roles for these receptor-genes both in disease and as therapeutic targets.

In humans, *FCRL6* encodes a type I surface glycoprotein with three Ig-like domains, a hydrophobic transmembrane segment, and a cytoplasmic tail with two tyrosines that constitute a consensus ITIM or a non-canonical ITAM (Table 1). *FCRL6* is also distinguished by its chromosomal position apart from the *FCRL1*-*5* locus and distribution outside the B cell lineage. *FCRL6* transcripts are expressed by mature NK cells and cytotoxic T cells in the adult spleen and blood [1,3,16]. In the circulation, the FCRL6 protein is detected on more differentiated cytotoxic NK cells possessing high levels of cytoplasmic perforin [12,18,19]. However, in early functional work, FCRL6 did not appear to markedly influence NK cell degranulation, CD16-mediated killing or cytokine production [19,20]. Immunoprecipitation studies identified the recruitment of the SHP-2 phosphatase to the FCRL6 cytoplasmic tail, uncovering its potential inhibitory function in cytotoxic lymphocytes [19,20]. Furthermore, the discovery of MHCII/HLA-DR as a ligand indicated the existence of interactions between FCRL6-expressing effector lymphocytes and antigen-presenting cells or other cell types that express MHCII/HLA-DR [11]. Accordingly, MHCII/HLA-DR molecules can be endogenously upregulated by tumor cells in cancer patients [21]. This expression pattern is associated with positive responses to anti-PD-1 immune checkpoint inhibitor (ICI) therapy in multiple cancer types, including ovarian cancer, classical Hodgkin’s disease, and melanoma [22,23,24]. Yet, MHCII+ cancers can become resistant to chronic ICI therapy, adapting and exploiting alternative checkpoint pathways, one of which may include suppressing T and NK cell effector responses via LAG3 and/or FCRL6, both of which can interact with MHCII/HLA-DR [25].

*CRL6* gene representatives are widely present in all mammalian families, including basal mammals such as elephants, pangolins, and armadillos. Armadillos belong to the Dasypodidae, the last extant family of the order [26,27,28,29,30,31,32,33]. Xenarthan placental mammals were first recorded among South American fossils in the Paleocene and are divided into three orders: the Cingulata, which includes armadillos; the Vermilingua, represented by anteaters; and the Phyllophaga suborder, that includes sloths [26,28,29,34]. Currently, there are 21 extant species of armadillos (Cingulata, Dasypodidae) [27,28,32,35,36,37,38]. These species primarily inhabit Central and South America, except for *D. novemcinctus*, which is more commonly known as the nine-banded armadillo and is found from Patagonia, Argentina, through to the southern United States [28,33,36]. As a result, *D. novemcinctus* is the most extensively spread Xenarthra species and has even been declared an invasive species by the US Department of Fish and Wildlife Services [28,33,36,38,39].

The armadillo has a number of characteristics that make it a relevant and useful research model. Most importantly, it exhibits susceptibility to leprosy, a human tropical disease mediated by the *Mycobacterium leprae* pathogen. Although this placental mammal has been defined as one of the best experimental models of leprosy, surprisingly, only about 5% of naturally infected animals develop clinical symptoms of the disease [40,41]. This observation suggests that *D. novemcinctus* may have undergone compensatory evolutionary immune adaption, resulting in the development of defense mechanisms that thwart *M. leprae* pathogenesis.

Here, we performed evolutionary analyses focused on the *FCRL6* gene in mammals. The unique pattern of gene duplication and diversification observed for *FCRL6* in *D. novemcinctus* is strongly suggestive of subfunctionalization, with intriguing relevance for the adaptation of this placental mammal in cell-mediated defense responses. These findings provide new insight into the immune system of this biological model and advance our understanding of the evolutionary importance of *FCRL* genes.

## 2. Results

### 2.1. Genomic Synteny Analysis of FCRL6 in Mammals

**Figure 1:**
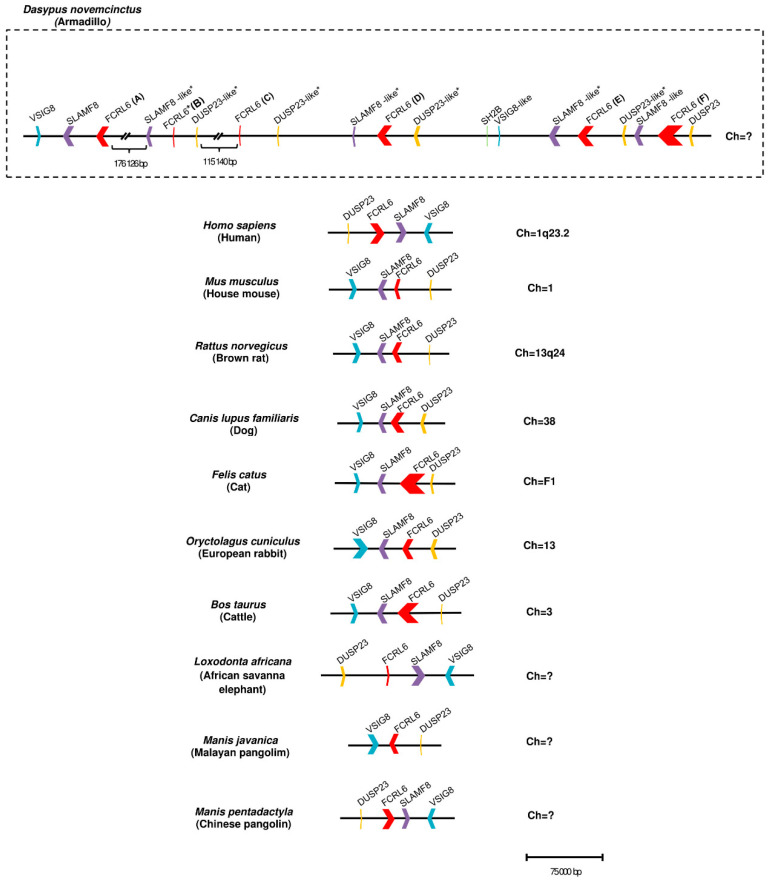
Structure of the *FCRL6* locus among 11 mammalian genomes. Horizontal lines correspond to the chromosome on which *FCRL6* and its flanking genes are located. Genes are color-coded: *FCRL6* (red), *SLAMF8* (purple), *VSIG8* (blue), *DUSP23* (yellow) and *SH2B* (green). Pseudogenes are labelled with an asterisk (*).

Compared to other *FCRL* family members, *FCRL6* has widespread representation in mammals. For example, *FCRL2* is absent in multiple genomes, including *M. musculus*, *R. norvegicus*, *B. taurus*, *B. bubalis*, *Eptesicus fuscus* and *Myotis lucifugus* [42]. However, an analysis of 21 mammalian genomes revealed that most possess a single copy of *FCRL6*, which is located in a conserved position between the *SLAMF8* and *DUSP23* genes (Figure 1). Surprisingly, we identified a massive block duplication of *FCRL6* and its neighboring genes (*SLAMF8* and *DUSP23*) in the *D. novemcinctus* genome. Figure 1 details five potentially functional copies of the *FCRL6* gene and one pseudogene. Table 2 lists these *D. novemcinctus FCRL6* gene replicates, which are listed A to F to simplify their nomenclature and relationships. Most copies, except for *FCRL6C*, are positioned with the *SLAMF8* gene to the left and the *DUSP23* gene to the right. They are also located in the telomeric region, a chromosomal region more prone to duplication, which supports the hypothesis of the block duplication of this genomic region. Despite the widespread representation of *FCRL6* among the genomes analyzed, we also found evidence for high structural diversity, with different species having between one and three Ig-like domain-encoding exons, as shown in Table 1.

### 2.2. Phylogenetic and Evolutionary Analysis of FCRL6 in D. novemcinctus

**Figure 2:**
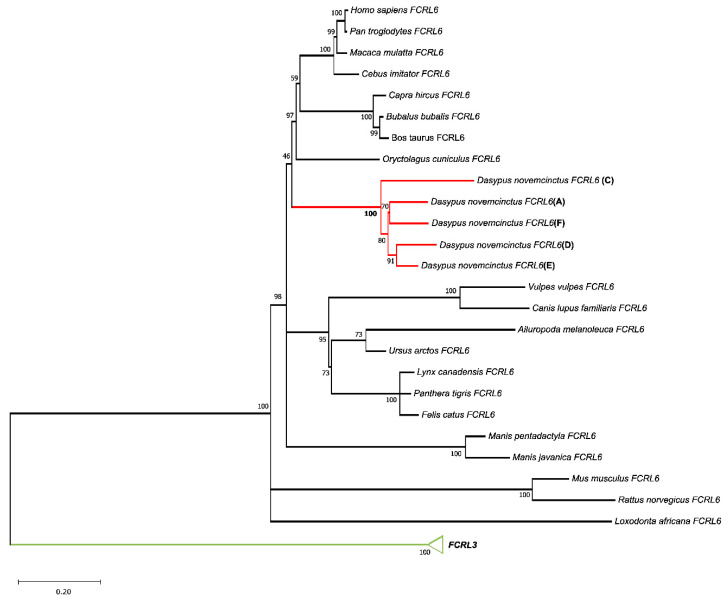
Phylogenetic analysis comparing representative mammalian and *D. novemcinctus FCRL6* cDNA sequences. The maximum likelihood (ML) method and the T92 + G + I model of nucleotide substitution were used to assemble the phylogenetic tree. Some groups were collapsed for simplification. The tree includes *FCRL6* genes from *D. novemcinctus* (*n* = 5) (red), *FCRL6* genes from other indicated mammals (*n* = 20) (black), and was rooted with *FCRL3* (*H. sapiens*, *B. taurus*, *O. cuniculus*, *F. catus* and *M. monocero*) (green). Bootstrap values are indicated to the left of each node.

We next analyzed the predicted *D. novemcinctus FCRL6* genes to determine whether they: (1) possess primary structures, namely, Ig-like domains, conserved with *FCRL6* representatives from other mammals, or (2) might be similar but different genes that resulted from the mutation, exon shuffling, or recombination of *FCRL6* with neighboring gene(s) in this locus. The analysis of the exons from these species also revealed the hallmark 21 bp exon that constitutes the second half of the split signal peptide that characterizes the FCR and *FCRL* families [3]. We next performed a phylogenetic analysis using the five predicted functional *D. novemcinctus FCRL6* cDNA sequences (copies **A**, **C**–**F**) identified by BLASTN, using a Maximum-Likelihood (ML) method and the T92 + G + I model of nucleotide substitution. The resulting ML phylogenetic tree supports the extensive duplication of the *FCRL6* gene in *D. novemcinctus*, as indicated by the distinct grouping of the five copies from a subtree branch relative to 20 other mammalian *FCRL6* representatives, with a bootstrap value of 100 (Figure 2). These findings indicate that the predicted *D. novemcinctus FCRL6* genes are, in fact, all *FCRL6* representatives. Collectively, these results support that all *FCRL6* genes found in *D. novemcintus* are *FCRL6* orthologs and are not distinct or related genes resulting from mutation, exon shuffling or recombination.

### 2.3. Protein Structure Analysis

**Figure 3:**
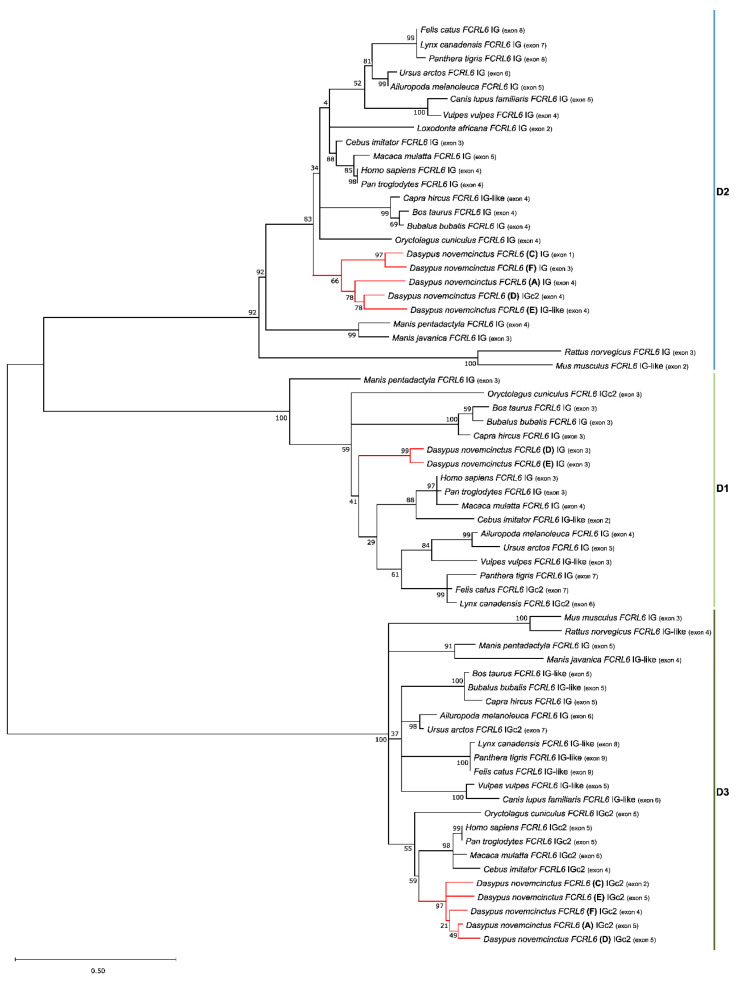
Phylogenetic tree of the predicted amino acid sequences for *FCRL6* Ig domains from *D. novemcinctus* and a panel of mammals (*n* = 20). The maximum likelihood (ML) method and the JTT + G + I model of amino acid substitution were used for analysis and assembly. D1 corresponds to the membrane-distal Ig-like domain, D2 to a middle Ig-like domain, D3 to a membrane-proximal Ig-like domain.

We next investigated the structural and phylogenetic relationships of *FCRL6* amino acid sequences predicted to encode type I proteins harboring Ig-like domains. An analysis of the five protein sequences from armadillo *FCRL6A* and *C*–*F* demonstrated similar interspecies structural variation to that previously observed in other mammals, including humans and rodents [3], with copies possessing either two or three Ig domains followed by a transmembrane region (Table 1). Specifically, *FCRL6A*, *C*, and *F* had two Ig-like domains, whereas *FCRL6D* and *E* had three. We then performed phylogenetic analyses of the amino acid sequences for these 12 *FCRL6* Ig-like domains. The generation of ML trees revealed evidence for three different domain subtypes (D1–D3) among the five *D. novemcinctus* gene copies that were clustered in independent branches (Figure 3).

All domains grouped with high bootstraps (D1-100, D2-92 and D3-100) and in a membrane-distal to membrane-proximal fashion, as previously described [1,3]. Thus, the *FCRL6* Ig-like domain subunits that are highly duplicated among the five gene copies in *D. novemcinctus*, but generally derive from single gene copies among most mammals, having evolved in a conserved manner as tandem exons-encoding domains in a similar membrane-proximal to -distal orientation. However, when we looked at the *D. novemcinctus* Ig-like domains, the D2 domains were grouped with the lowest bootstrap values. This prompted us to analyze the mean distance within each domain (Table 3). This approach disclosed that the D2 domain has the highest diversity compared to the other two domain types, sharing only 75.4% of the identity. Notably, this finding is in contrast to prior observations that *FCRL* Ig-like domains most distal to the transmembrane region generally have higher diversity and lower sequence identity [1,3].

An examination of the predicted intracellular regions of *FCRL6A* and *C*–*F* revealed that four out of the five copies had cytoplasmic tails, but *FCRL6C* did not. Prediction software indicated that it was unlikely that this representative is tethered to the plasma membrane by a glycosylphosphatidylinositol (GPI) anchor and glypiation [43]. To characterize the potential cellular location of *FCRL6* copies in *D. novemcinctus*, each sequence was analyzed using the DeepLoc 2.0 server (Table 4) [44]. All *FCRL6* copies appeared to be localized in the cell membrane, even the shorter *FCRL6C* representative.

**Figure 4:**
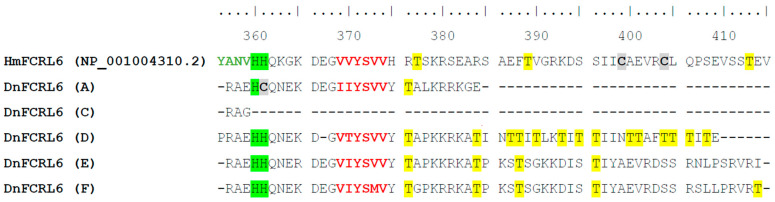
Multiple sequence alignment of the human and nine-band armadillo *FCRL6* cytoplasmic tails. Marked are potential tyrosine-based activating and inhibitory sequences, along with cysteine, histidine and threonine residues. Alignment was performed using CLUSTALW in the BioEdit software package. An ITAM-like sequence (*H. sapiens*) is colored green and ITIM are red. Cysteines are in black, bold and highlighted grey. Histidine is highlighted green. Threonine is highlighted yellow.

Closer evaluation of the *FCRL6A* and *D*–*F* proteins showed the presence of potential cytoplasmic tyrosine-based motifs. Alignment of these sequences showed evidence of conservation with the human *FCRL6* cytoplasmic tail, but remarkable inter-copy variation. An assessment of tyrosine-based ITIM (I/V/L-x-Y-x-x-L/V) or ITAM (D/Ex-x-Y-x-x-L/I-x6–8-Y-x-x-L/I) sequences [1,45,46], disclosed that these four representatives with long tails possessed consensus ITIM (Figure 4). However, while all sequences, have two histidine residues in the position 360 and 361, the *D. novemcinctus* A copy has one histidine replaced by a cysteine. Another peculiarity is found in copy D, where a glutamate (negatively charged) was lost and, instead of the 3 to 5 residues of threonine (polar and uncharged residue), 15 are present.

The number of extracellular cysteine residues present in the *D. novemcinctus FCRL6* copies is mostly consistent (six to eight residues), with the exception of the C copy, which only has four residues. However, previous studies have determined that the human *FCRL6* cysteines are unlikely to be involved in the homodimerization of the protein, so it is unclear if this intracellular cysteine has the potential to change the tridimensional conformation of this *D. novemcinctus* protein and affect its ability to be membrane-bound or segregated [20].

## 3. Discussion

Here, we identify evidence for marked *FCRL6* expansion, duplication and subfunctionalization in *D. novemcinctus*. Among mammals, *FCRL6* exhibits marked interspecies genetic variation (highlighted in Table 1). For example, the mouse and rat representatives have only two Ig domains and lack consensus ITIM or ITAM [3,47]. Despite these structural differences, the genomic synteny of the *FCRL6* locus among mammalian lineages was largely maintained. In *D. novemcinctus*, this conserved synteny appears to be repeated in a three-gene block, with six copies of the *FCRL6* gene each flanked on the left by *SLAMF8* and on the right by *DUSP23*. One exception is *FCRL6A*, which is missing *DUSP23* on the right, and *FCRL6C*, which is missing *SLAMF8* on the left. Among these gene copies, only five appear to be functional, with one three-gene block replicate completely pseudogenized. Examining its chromosomal position among major mammalian families, it is evident in *Homo sapiens*, *Mus musculus*, *Canis lupus familiaris*, *Felis catus* and *Bos taurus* that the *FCRL6* gene is always closer to the telomere, a region known to be rich in gene duplication [48]. Gene duplication is one of the driving forces of evolution and paralogs can either gather deleterious mutations, become subfunctionalized or become neofunctionalized [48]. The fact that *Loxodonta africana* has only one *FCRL6* gene copy suggests that a duplication event did not occur in the Atlantogenata sister group, and thus may be exclusive to Xenarthra. However, based on the available genome sequences, all other Xenarthra species (*Choloepus hoffmanni* and *Choloepus didactylus*) seem to possess only one copy of the gene. Intriguingly, only single copies of *SLAMF8* and *DUSP23* at the distal ends of the locus appear to retain functionality, with all other replicates pseudogenized. Thus, this mass duplication of five potentially functional copies of *FCRL6* appears to be exclusive to the *D. novemcinctus* species and could have marked functional impact.

Phylogenetic analysis at the cDNA level was revealing because the five *FCRL6* copies in the armadillo were not only grouped with the other mammalian representatives, which lends support to the duplication hypothesis, but branched together independently. This latter finding is consistent with the subfunctionalization hypothesis for these five paralogous genes. At the protein level, phylogenetic analysis of the 12 *FCRL6* Ig-like domains disclosed their relatedness with three mammalian *FCRL6* Ig domain subtypes (D1–D3) by grouping in a conserved pattern according to their relative distance from the cell membrane. This further supports the hypothesis that these genes maintain functionality and may have acquired additional subfunctions during their evolution. However, in contrast to previous findings of membrane-distal *FCRL* Ig-like domains possessing the highest sequence diversity [2], in *D. novemcinctus*, D2 domains displayed the greatest diversity. This may indicate that the ligands for these receptors in this species are polymorphic.

Given that each *FCRL6* copy possesses characteristic amino-terminal Ig domains, transmembrane regions and carboxy-terminal cytoplasmic tails, they are predicted to be type I membrane proteins. While the armadillo *FCRL6C* copy is annotated as a pseudogene in the NCBI database, we were able to translate and align its Ig-like domain sequences. Since it is the most divergent *FCRL6* gene copy and located the most proximally to the pseudogenized copy, it is possible that *FCRL6C* is in the process of pseudogenization. This could explain its incomplete sequence in the Ensemble database. However, this replicate still retains the major characteristics that define *FCRL6* receptors, despite lacking a cytoplasmic tail. Moreover, prediction software indicated that *FCRL6C* is most likely to be located in the cell membrane and not GPI-anchored.

Importantly, the *D. novemcinctus FCRL6A* and *D*–*F* copies all possess two cytoplasmic tyrosines. One of these tyrosine residues aligns with human *FCRL6* and comprises a canonical ITIM. This finding indicates that, like human *FCRL6*, these four copies in *D. novemcinctus* could possess the capacity to recruit phosphatases that exert suppressive cellular function. Another notable intracellular feature in the A copy includes the change of a histidine residue to a cysteine, which might modify the tridimensional conformation or signaling properties of the cytoplasmic tail. Moreover, the D copy has an overrepresentation of cytoplasmic threonines, enriching a reactive amino acid that can establish hydrogen bonds with a large number of polar substrates and can be involved in phosphorylation.

These findings of four potential ITIM-bearing *FCRL6* receptors in *D. novemcinctus* have led to the hypothesis that this marked expansion might impact immunoregulatory suppression and host tolerance to certain pathogens in these mammals. In humans, *FCRL6* is expressed by effector T and NK lymphocytes and binds HLA-DR/MHCII on antigen-presenting cells (APC) such as B cells, dendritic cells, and macrophages [11]. Importantly, the nine-banded armadillo is known to be asymptomatic when infected by the intracellular parasite *Trypanosoma cruzi*, which enters macrophages during certain stages of its life cycle [49]. A second example is *M. leprae*, which also infects macrophages and dendritic cells and can produce up to 100 bacilli per cell [41,50]. However, armadillos have few discernible cutaneous signs of leprosy, with 15–20% of experimentally inoculated individuals being resistant and wild individuals in Texas, Florida and Mississippi having a rate of infection of between 0% and 29% [41,51]. Cytotoxic T cells have the ability to lyse APCs that are infected by *M. leprae* through the use of perforin and granzyme, which form pores in target cells and activate caspases that induce cell death [41,52]. NK cells are also recruited to leprosy lesions by IL-2, where they can clear and eliminate infected macrophages and Schwann cells [41]. Given the constrained pathogenicity of these two organisms, it is tempting to speculate that the additional *FCRL6* copies evident in *D. novemcinctus* may confer this species tolerization against certain intracellular pathogens that invade APCs. If *FCRL6* maintains a conserved expression pattern by effector lymphocytes in the armadillo, such expanded ITIM-mediated suppression in T and NK cells could curb cytotoxicity against the MHCII-expressing APCs that harbor these pathogens.

Evidence of a role for *FCRL6* in tolerance and immune-evasion also comes from the tumor immunity. Studies of human solid tumors reveal that, similar to LAG3, *FCRL6* is upregulated in malignancies that are MHCII/HLA-DR+ [12,25]. An analysis of the Cancer Genome Atlas (TCGA) RNA-sequencing data from non-hematopoietic cancer types disclosed that elevated *FCRL6* expression in the tumor microenvironment correlates with improved overall survival and progression-free survival in melanoma, breast cancer and non-small cell lung cancer [12]. However, despite this favorability, some tumors may alternatively upregulate HLA-DR expression to blunt recognition by *FCRL6*-expressing cytotoxic cells and other immunoreceptors, as a strategy to tolerize effector lymphocyte responses and resist recognition and clearance [21]. Thus, further in vivo studies are required to better understand *FCRL6* regulation and function in these contexts. However, this is somewhat complicated by its diversity among species, including humans and mice [12].

In summary, these newfound features of the *FCRL6* immunoreceptor highlight the relevance of its interspecies divergence and evolutionary history. The identification of this distinct evolutionary event in *D. novemcinctus* should aid our understanding and hopefully uncover new functions for the *FCRL6* receptor, especially with regard to its inhibitory function, role as a potential checkpoint target in tumor immunity and its involvement in host defense responses. These observations collectively imply a special role for *FCRL6* at the interface of immune tolerance and defense.

## 4. Materials and Methods

**Sequences.** Mammalian *FCRL6* gene sequences were obtained from Ensembl (https://www.ensembl.org/index.html (accessed on 10 November 2021)) and the NCBI GenBank (http://www.ncbi.nlm.nih.gov/genbank/ (accessed on 10 November 2021) [42,53]. The sequences were aligned using ClustalW [54] and BioEdit [55] software to identify intron/exon boundaries and open reading frames. The resulting alignments are available in the Supplementary Material (Files S1 and S2). Overall, 24 *FCRL6* sequences from 20 species representative of the Primata, Rodentia, Lagomorpha, Artiodactyla, Carnivora and Proboscidea orders were analyzed (accession numbers are provided in Supplementary Table S1). The sequences that had frameshift deletions or insertions or/and premature stop codons were excluded.

**Genomic Synteny Analysis.** A map drawn to the approximate scale of the loci, including the inter- and intra-genic distances, was constructed to demonstrate the relative syntenic positions among genomes. Gene loci were determined using the Ensembl (https://www.ensembl.org/index.html (accessed on 10 November 2021) and NCBI (https://www.ncbi.nlm.nih.gov/gene/ (accessed on 10 November 2021) databases (provided in Supplementary Table S2).

**Protein Structures.** The SMART (Simple Modular Architecture Research Tool) web resource (http://smart.embl.de (accessed on 1 July 2022)), which interfaces with the UniProt, Ensembl and STRING protein databases, was used to identify and annotate *FCRL6* protein domains for individual species [56,57]. To determine whether *D. novemcinctus FCRL6* sequences were GPI-anchored, the NetGPI 1.1 [43] web tool (https://services.healthtech.dtu.dk/service.php?NetGPI-1.1 (accessed on 2 December 2022)) was used. This tool uses a deep learning approach, which is based on recurrent neural networks to predict glycosylphosphatidylinositol anchoring (GPI-anchoring or glypiation). To confirm if *FCRL6* copies were present in the cell membrane, the DeepLoc 2.0 [44] web tool (https://services.healthtech.dtu.dk/service.php?DeepLoc (accessed on 2 December 2022)) was used. DeepLoc 2.0 is able to predict the subcellular localization of eukaryotic proteins using a Neural Networks algorithm trained on Uniprot proteins. To ensure the accuracy of the predictions, both full-length and exon-derived amino acid sequence segments were analyzed.

**Phylogenetic Analysis.** To determine the phylogenetic relationships between *FCRL6* genes, MEGA version X 10.2.6 software [58] with a Maximum Likelihood (ML) framework was used. The Model Selection option in MEGA version X was used to determine the best fitting model for our datasets. For the analysis of full-length *FCRL6* nucleotide sequences, the T92 + G + I model was used to construct the phylogenetic tree. To root the tree, *FCRL3* sequences of *Homo sapiens*, *Bos taurus*, *Oryctolagus cuniculus*, *Felis catus* and *Monodon monoceros* were included, and node support was estimated using 1000 bootstrap replicates of ML trees. For the Ig-like domain amino acid analysis, an ML framework and the JTT + G model were applied to establish the relationship between the different domains of the sequences, with node support being estimated using 1000 bootstrap replicates of ML trees. The mean distances within Ig-like domain groups and between Ig-like domain groups were calculated using MEGAX.

## 5. Conclusions

This study brought to light the extensive duplication of the *FCRL6* gene, which appears to be exclusive to the nine-banded armadillo. The synteny of this genomic location is preserved among mammals and the *FCRL6* copies retain a conserved structure, which implies that these proteins might have undergone subfunctionalization as a result of their massive duplication. Importantly, four copies possess consensus ITIM in their cytoplasmic tails, but the D copy has an excess of threonine and the A copy has a histidine residue replaced by a cysteine, which may have important functional implications for the inhibitory properties of these receptors. The fact that this duplication is distinct to *D. novemcinctus*, which possesses symptomatic resistance to intracellular pathogens such as leprosy, has implications for host defense and tolerance. The hypothesis that an expansion of *FCRL6* inhibitory receptor copies in armadillo effector lymphocytes may enhance host suppression, by binding MHCII-expressing antigen-presenting cells that are infected by *M. leprae* and curbing responses, requires further functional and expression studies.

## Figures and Tables

**Table 1:** Predicted domains, repeats, motifs and features of Fc receptor-like 6 (FCRL6) among a panel of mammalian species (http://smart.embl-heidelberg.de/ (accessed on 1 July 2022)). IG, immunoglobulin domain (green); IGc2, immunoglobulin C-2 type domain (dark green); IG-like, Immunoglobulin-like (light green); transmembrane region (dark blue rectangle); low complexity region (light blue rectangle).

Species	FCRL6				
**Armadillo**	**A**	**C**	**D**	**E**	**F**
	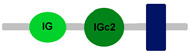	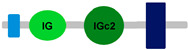	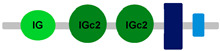	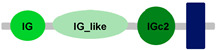	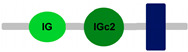
**Human**	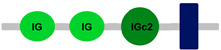	**-----------------------**	**-----------------------**	**-----------------------**	**-----------------------**
**House mouse**	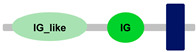	**-----------------------**	**-----------------------**	**-----------------------**	**-----------------------**
**Brown rat**	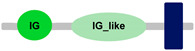	**-----------------------**	**-----------------------**	**-----------------------**	**-----------------------**
**Dog**	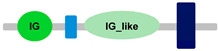	**-----------------------**	**-----------------------**	**-----------------------**	**-----------------------**
**European rabbit**	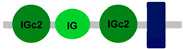	**-----------------------**	**-----------------------**	**-----------------------**	**-----------------------**
**Cattle**	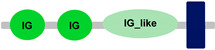	**-----------------------**	**-----------------------**	**-----------------------**	**-----------------------**
**Cat**	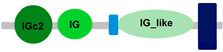	**-----------------------**	**-----------------------**	**-----------------------**	**-----------------------**
**Malayan pangolin**	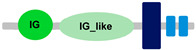	**-----------------------**	**-----------------------**	**-----------------------**	**-----------------------**
**Chinese pangolin**	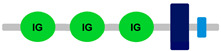	**-----------------------**	**-----------------------**	**-----------------------**	**-----------------------**

**Table 2:** Proposed nomenclature for *FCRL6* gene copies in *D. novemcinctus*.

Proposed Nomenclature	Gene Symbol	DNA Accession Number	Protein Accession Number
A	LOC101434982	XM023588041.1	XP_023443809.1
B (pseudogenized)	LOC105745885	-------------------------	-------------------------
C	ENSDNOG00000034278	ENSDNOT00000037015.1	ENSDNOP00000030820
D	LOC105745887	XM023588042.1	XP_023443810.1
E	LOC101436671	XM023588037.1	XP_023443805.1
F	LOC101437529	XM012524200.1	XP_012379654.1

**Table 3:** Mean within-group distances (D1, D2 and D3) of the *D. novemcinctus* Ig-like ectodomains calculated in MEGAX.

Group	Mean Distance
D1	0.124
D2	0.246
D3	0.145

**Table 4:** Subcelullar localizations of *FCRL6A* and *C*–*F* in *D. novemcinctus* predicted by DeepLoc 2.0.

	Location									
Isoform	Cytoplasm	Nucleus	Extracellular	Cell Membrane	Mitochondrion	Plastid	Endoplasmic Reticulum	Lysosome/ Vacuole	Golgi Apparatus	Peroxisome
A	0.3008	0.1125	0.1564	**0.8483**	0.0402	0.0032	0.2972	0.3723	0.3315	0.1876
C	0.2555	0.1032	0.2319	**0.8221**	0.0621	0.0065	0.4657	0.2457	0.3461	0.1602
D	0.2764	0.0838	0.1796	**0.8735**	0.0482	0.0018	0.3150	0.3218	0.3190	0.1497
E	0.2943	0.0979	0.1463	**0.8273**	0.0380	0.0018	0.3075	0.3133	0.3356	0.1138
F	0.2563	0.0838	0.1672	**0.8512**	0.0272	0.0030	0.2597	0.3118	0.3098	0.1463

## Data Availability

The dataset analysed in this study is available as supplementary material.

## Supplementary Materials

The supporting information can be downloaded at: https://www.mdpi.com/article/10.3390/ijms24054531/s1.
