# Supplementary material for: Evidence for Extensive Duplication and Subfunctionalization of FCRL6 in Armadillo (Dasypus novemcinctus)
Source: Int J Mol Sci. 2023 Feb 25;24(5):4531. doi: 10.3390/ijms24054531 (PMC10003336; doi:10.3390/ijms24054531)
Supplement: Supplementary file 1 [file ijms-24-04531-s001.zip › ijms-2184098-supplementary Tables.pdf]

International Journal of *Molecular Sciences*

MDPI

Supplementary Material

Table S1. Genbank accession number of the sequences used in this study

Species	Common name	Order-Family	Accession number	Data base annotation
Homo sapiens	Human	Primates – Hominidae	NM_001004310.3	Fc receptor-like 6
Pan troglodytes	Chimpanzee	Primates – Hominidae	XM_016930149.2	Fc receptor-like 6
Macaca mulata	Rhesus monkey	Primates – Cercopithecidae	XM_015112823.2	Fc receptor-like 6
Cebus imitator	Panamanian white-faced capuchin	Primates – Cebidae	XM_037729642.1	Fc receptor-like 6
Capra hircus	Goat	Artiodactyla	XM_005677243.3	Fc receptor-like 6
Bubalus bubalis	Water buffalo	Artiodactyla	XM_006061019.4	Fc receptor-like 6
Bos taurus	European cattle	Artiodactyla – Bovidae	XM_015461679.2	Fc receptor-like 6
Oryctolagus cuniculus	European rabbit	Lagomorpha – Leporidae	XM_017345767.1	Fc receptor-like 6
Dasypus novemcinctus	Nine-banded armadillo	Xenarthra – Dasipodidae	XM_023588041.1	Fc receptor-like 6
Dasypus novemcinctus	Nine-banded armadillo	Xenarthra – Dasipodidae	ENSDNOT00000037015.1	Novel gene
Dasypus novemcinctus	Nine-banded armadillo	Xenarthra – Dasipodidae	XM_023588042.1	Fc receptor-like 6
Dasypus novemcinctus	Nine-banded armadillo	Xenarthra – Dasipodidae	XM_023588037.1	Fc receptor-like 6
Dasypus novemcinctus	Nine-banded armadillo	Xenarthra – Dasipodidae	XM_012524200.1	Fc receptor-like 6
Vulpes vulpes	Red fox	Carnivora – Canidae	XM_025986362.1	Fc receptor-like 6
Canis lupus familiaris	Dog	Carnivora – Canidae	ENSCAFT00845055076.1	Fc receptor-like 6
Ailuropoda melanoleuca	Giant panda	Carnivora – Canidae	XM_011217660.3	Fc receptor-like 6
Ursus arctos	Brown bear	Carnivora – Canidae	XM_026487349.3	Fc receptor-like 6

Lynx canadensis	Canada lynx	Carnivora – Felidae	XM_032591719.1	Fc receptor-like 6
Panthera tigris	Tiger	Carnivora – Felidae	XM_042975722.1	Fc receptor-like 6
Felis catus	Domestic cat	Carnivora – Felidae	XM_023247467.1	Fc receptor-like 6
Manis pentadactyla	Chinese pangolin	Pholidota, Manidae	XM_036922675.1	Fc receptor-like 6
Manis javanica	Malayan pangolin	Pholidota, Manidae	XM_037023917.1	Fc receptor-like 6
Mus musculus	House mouse	Rodentia – Muridae	DQ470838.1	Fc receptor-like 6
Rattus norvegicus	Norway rat	Rodentia – Muridae	NM_001164726.1	Fc receptor-like 6
Loxodonta africana	African savanna elephant	Afrotheria, Proboscidea	XM_023553949.1	Fc receptor-like 6
FCRL3				
Homo sapiens	Human	Primates – Hominidae	NM_052939.4	Fc receptor like 3 (FCRL3)
Bos Taurus	European cattle	Artiodactyla – Bovidae	XM_024989896.1	Fc receptor like 3
Monodon monoceros	Narwhal	Cetacea – Monodontidae	XM_029227202.1	Fc receptor like 3 (FCRL3)
Felis catus	Cat	Carnivora – Felidae	XM_023247510.2	Fc receptor like 3
Oryctolagus cuniculus	European rabbit	Lagomorpha – Leporidae	ENSOCUT00000009035.4	Fc receptor like 3

International Journal of Molecular Sciences

MDPI

Table S2. Genome assembly and gene location for the sequences used to construct the synteny maps.

Species	Common name	Genome assembly	Chromosome	Location
Dasypus novemcinctus	Nine-banded armadillo	Dasnov3.0 (GCF_000208655.1)	Unplaced Scaffold	NW_004474876.1 (63589..76081, complement)
Homo sapiens	Human	GRCh38.p14 (GCF_000001405.40)	1	NC_000001.11 (159800512..159816257)
Mus musculus	House mouse	GRCm39 (GCF_000001635.27)	1	NC_000067.7 (172423081..172445889, complement)
Rattus norvegicus	Norway rat	mRatBN7.2 (GCF_015227675.2)	13	NC_051348.1 (85062709..85072308, complement)
Canis lupus familiaris	Dog	ROS_Cfam_1.0 (GCF_014441545.1)	38	NC_051842.1 (22433000..22442314, complement)
Felis catus	Cat	F.catus_Fca126_mat1.0 (GCF_018350175.1)	F1	NC_058384.1 (64740117..64749544, complement)
Oryctolagus cuniculus	European rabbit	OryCun2.0 (GCF_000003625.3)	13	NC_013681.1 (33312755..33323526, complement)
Bos taurus	Cattle	ARS-UCD1.2 (GCF_002263795.1)	3	NC_037330.1 (9870119..9891238, complement)
Loxodonta africana	African savanna elephant	Loxafr3.0 (GCF_000001905.1)	Unplaced Scaffold	NW_003573453.1 (9153935..9156948)
Manis javanica	Malayan pangolim	YNU_ManJav_2.0 (GCF_014570535.1)	Unplaced Scaffold	NW_023436095.1 (7718134..7738591, complement)
Manis pentadactyla	Chinese pangolin	YNU_ManPten_2.0 (GCF_014570555.1)	Unplaced Scaffold	NW_023456141.1 (6491033..6507646)
